# Effects of Astragaloside IV Combined With Metronidazole on Cyst Shedding and Mucosal Immune and Inflammatory Responses in a Murine Model of Giardiasis

**DOI:** 10.1155/japr/1200923

**Published:** 2026-08-17

**Authors:** Hossein Mahmoudvand, Amal Khudair Khalaf, Sahar Mahmoudvand, Ahmad Adineh, Aram Oladi, Javad Ghasemian Yadegari

**Affiliations:** ^1^ Razi Herbal Medicines Research Center, Lorestan University of Medical Sciences, Khorramabad, Iran, lums.ac.ir; ^2^ Department of Microbiology, College of Medicine, University of Thi-qar, Thi-qar, Iraq, utq.edu.iq; ^3^ Department of Toxicology, Lorestan University of Medical Sciences, Khorramabad, Iran, lums.ac.ir; ^4^ Department of Pharmacognosy, Lorestan University of Medical Sciences, Khorramabad, Iran, lums.ac.ir

**Keywords:** giardiasis, in vivo, inflammation, natural products, treatment

## Abstract

**Background:**

Giardiasis continues to be a significant intestinal parasitic infection, with instances of treatment failure and recurrence underscoring the necessity for supplementary therapeutic approaches. Astragaloside (AGS), a bioactive compound known for its anti‐inflammatory and immunomodulatory effects, may serve as a promising adjunctive treatment; however, its efficacy against *Giardia* infection has not been thoroughly examined. Accordingly, this study aimed to assess the antigiardial efficacy of AGS both as a monotherapy and in combination with metronidazole (METR), focusing specifically on parameters such as parasite cyst burden and viability, intestinal mucosal immune responses, inflammatory markers, and hepatic safety associated with treatment.

**Methods:**

The MTT colorimetric assay was used to evaluate the in vitro antigiardial effects of AGS against *Giardia* trophozoites. Mice infected with *Giardia* were treated with AGS (10 and 15 mg/kg/day) and METR (7.5 and 15 mg/kg/day), both individually and in combination, over a 7‐day period. Subsequently, stool samples were collected and analyzed to assess the presence and viability of *Giardia* cysts. The levels of secretory IgA (sIgA) in intestinal tissue, as well as the expression of tumor necrosis factor‐alpha (*TNF-α*), nuclear factor kappa B (*NF-κB*), and interleukin‐1 beta (*IL-1β*), were quantified using commercial ELISA kits and real‐time polymerase chain reaction (PCR), respectively.

**Results:**

AGS exhibited a clear concentration‐dependent inhibitory effect on the viability of *Giardia* trophozoites, with a calculated half‐maximal inhibitory concentration (IC_50_) value of 30.24 ± 2.43 *μ*g/mL. The administration of either METR or AGS (10 and 15 mg/kg) individually resulted in a partial reduction in both cyst quantity and viability. Notably, combined treatment with AGS and METR resulted in no detectable cysts in fecal samples collected 24 h after the final treatment dose, within the detection limits of microscopy‐based analysis (*p* < 0.001). Secretory IgA concentrations showed modest improvement following monotherapy but increased substantially after combination therapy (4.36 ng/mL, *p* < 0.001). Correspondingly, elevated levels of pro‐inflammatory markers *TNF-α*, *NF-κB*, and *IL-1β* observed in untreated mice (5.3‐, 4.29‐, and 5.16‐fold, respectively) were nearly restored to baseline values by the co‐administration regimen of AGS 15 mg/kg + METR 7.5 mg/kg (1.11‐, 0.86‐, and 0.91‐fold change, *p* < 0.001). These findings indicate that AGS not only lacks hepatotoxicity but may also exert protective effects against infection‐associated hepatic dysfunction.

**Conclusion:**

AGS administration was associated with a reduced *Giardia* cyst burden at the prespecified 24‐h post‐treatment endpoint, restoration of mucosal immune responses, attenuation of inflammatory signaling, and improved biochemical profiles in experimentally infected mice. These findings suggest that AGS may function as an immunomodulatory and anti‐inflammatory adjunct, potentially contributing to host‐mediated parasite load reduction and improved treatment outcomes when combined with METR. However, additional antiparasitic mechanisms and formal pharmacodynamic synergistic interactions were not demonstrated in this study and require further investigation through dedicated in vitro and mechanistic analyses.

## 1. Introduction

Giardiasis, caused by the flagellated protozoan *Giardia lamblia*, remains one of the most prevalent intestinal parasitic diseases worldwide, with an estimated 280 million new cases annually [1]. Transmission is highest in tropical and temperate regions, and in Iran, the northern provinces are considered endemic [2]. Infection occurs mainly through ingestion of cysts in contaminated water or food, or via fecal‐oral spread between individuals; children are disproportionately affected because of behavioral and immunological factors [3]. The disease ranges from a self‐limiting acute illness to a chronic condition marked by persistent diarrhea and malabsorption, which can cause growth retardation and long‐term nutritional deficits in young patients [2, 3].

Nitroimidazoles and nitrofurans, notably metronidazole (METR) and furazolidone, remain the mainstay of antigiardial therapy [4], but their clinical utility is limited by variable efficacy, adverse effects, and mutagenic or carcinogenic potential that restricts their use in pregnant women and children [5]. Rising reports of treatment failure further point to the emergence of drug‐resistant *Giardia* isolates [4, 5]. These constraints have intensified interest in plant‐derived antigiardial agents, valued for their availability, low cost, and favorable safety profiles [6]; several phytochemicals have shown antigiardial activity in experimental models [7], although inconsistent methodologies and a scarcity of in vivo efficacy data have limited their translational potential. Saponins, amphiphilic glycosides composed of a hydrophobic aglycone and hydrophilic sugar moieties, are abundant in medicinal plants and display antimicrobial, antiparasitic, anti‐inflammatory, immunomodulatory, and anticancer properties [8, 9]. Astragaloside (AGS), a cycloartane‐type triterpenoid saponin from Astragalus species, is considered a principal bioactive constituent underlying these effects and has demonstrated immunomodulatory, anti‐inflammatory, antioxidant, cardioprotective, neuroprotective, and hepatoprotective activity across multiple models [9, 10]. Its favorable oral tolerability and capacity to modulate NF‐*κ*B and inflammasome‐associated signaling—pathways central to *Giardia*‐induced intestinal inflammation—motivated its selection for the present study [10, 11].

We hypothesized that AGS would enhance mucosal immune responses and suppress nuclear factor kappa B (NF‐*κ*B)‐mediated inflammation, thereby enhancing the efficacy of METR in *Giardia*‐infected mice. The primary aim of this study was therefore to evaluate the effect of AGS, alone and combined with METR, on cyst burden and cyst viability in experimentally infected mice. Secondary aims included assessing intestinal mucosal immunity via secretory IgA levels, quantifying the inflammatory mediators tumor necrosis factor‐alpha (TNF‐*α*), NF‐*κ*B, and interleukin‐1 beta (IL‐1*β*), measuring serum electrolyte changes, and evaluating hepatoprotective effects through biochemical liver function markers.

## 2. Material and Methods

### 2.1. Ethical Compliance

All animal‐based experimental procedures were conducted in accordance with internationally accepted ethical standards for laboratory animal research, as outlined by the National Institutes of Health (NIH). Before the initiation, the study design and experimental protocols were thoroughly reviewed and approved by the Research Ethics Committee of Lorestan University of Medical Sciences (Khorramabad, Iran), under approval code IR.LUMS.REC.1404.339.

### 2.2. Isolation and Preparation of *G. lamblia* Cysts

Fecal specimens were collected from a clinically confirmed case of giardiasis admitted to Shahid Rahimi Hospital, Khorramabad. Initial identification of *G. lamblia* cysts was performed using direct microscopic examination combined with the formalin–ether concentration technique. To obtain highly purified cysts, samples underwent sucrose density gradient centrifugation (0.85 M), following previously validated protocols [12]. The purified cyst suspension was then standardized to a final concentration of 1 × 10^4^ cysts/mL.

### 2.3. In Vitro Antigiardial Screening of AGS on *G. lamblia* Trophozoites

Purified cysts of *G. lamblia* were subjected to an in vitro excystation protocol adapted from the previously validated excystation approach [13, 14]. Specifically, a suspension of purified cysts was combined with hydrochloric acid (HCl; 0.01 N, pH 2.0) at a ratio of 1:9 (v/v) and incubated at 37°C for 1 h to facilitate excystation. The sample was then centrifuged (600 rpm, 10 min), the supernatant discarded, and the resulting pellet transferred into sterile‐filtered TYI‐S‐33 medium containing bovine bile salts (Merck, Germany), 20% heat‐inactivated fetal calf serum (Merck, Germany), and antibiotics (penicillin 500 IU/mL, streptomycin 500 *μ*g/mL). Cultures were then propagated at 37°C in slanted tubes. Trophozoite suspensions (1 × 10^5^ cells/mL) were plated separately across 96‐well plates at 100 *μ*L per well. Wells were then treated with a concentration series (2–200 *μ*g/mL) of AGS (> 98% purity, Sigma‐Aldrich, Germany) or METR (Sigma‐Aldrich, Germany) and maintained at 37°C for 48 h. Supernatants were subsequently aspirated, and 20 *μ*L of MTT reagent (0.5 mg/mL, PBS‐diluted) was added to each well, followed by a 4‐h incubation under 5% CO₂ at 37°C. Formazan crystals were solubilized with 100 *μ*L dimethyl sulfoxide, and optical density was read at 570 nm on an ELISA microplate reader. IC_50_ values were derived through Probit regression modeling in SPSS (v.26.0) [13, 14]. Each experimental condition was evaluated in triplicate to increase measurement reliability and enhance the reproducibility of the assay.

### 2.4. Experimental Protocol for Murine Giardiasis

#### 2.4.1. Animal Model

Sixty‐four male BALB/c mice (8–10 weeks old; body weight 25–30 g) were used in this investigation. Animals were housed under controlled environmental conditions, including constant temperature, regulated humidity, and a 12 h light/dark cycle. Standard rodent feed and drinking water were made freely available throughout the study. Animals were randomly allocated into experimental groups using a computer‐generated randomization sequence. Investigators responsible for cyst counting, ELISA measurements, and qPCR analyses were blinded to treatment allocation during data acquisition and analysis.

#### 2.4.2. Infection Protocol

To increase intestinal susceptibility to parasite colonization, mice underwent a 4‐day antibiotic conditioning regimen prior to infection. During this period, their drinking water was supplemented with vancomycin (0.5 mg/mL), neomycin (1 mg/mL), and ampicillin (1 mg/mL). The animals were then orally inoculated with 0.2 mL of a suspension containing 2 × 10^3^ G*. lamblia* cysts per milliliter. Infection was confirmed by fecal microscopy on Day 6 post‐inoculation. Successful infection was confirmed by detecting cysts in fecal samples using direct smear microscopy and the formalin–ether concentration method [13].

#### 2.4.3. Treatment Allocation

**Figure 1:**
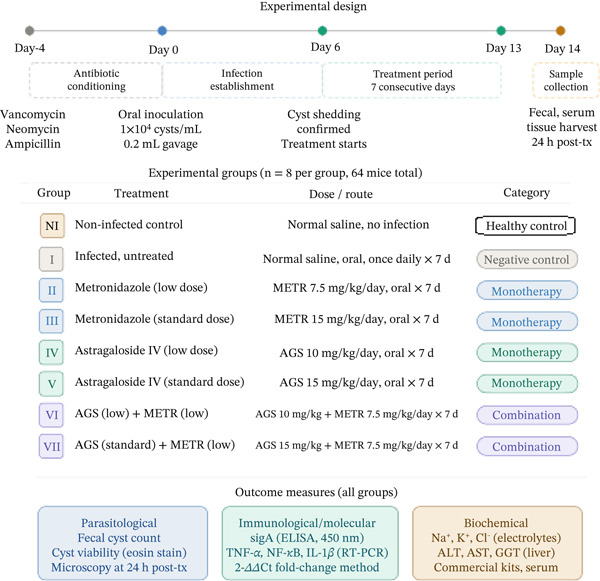
Schematic overview of the experimental design in the murine Giardia lamblia model. The timeline illustrates antibiotic conditioning, *G. lamblia* infection, treatment allocation, and sample collection. Mice were treated for seven consecutive days following infection confirmation, and fecal, serum, and intestinal tissue samples were collected after the final treatment. AGS: Astragaloside; ALT: alanine aminotransferase; AST: aspartate aminotransferase; GGT: gamma‐glutamyl transferase; METR: metronidazole; NI: noninfected control; sIgA: secretory immunoglobulin A.

Treatments were initiated 6 days post‐infection following confirmation of cyst shedding in fecal samples and were administered orally once daily at the same time each day for seven consecutive days. Mice were randomly assigned into eight groups (*n* = 8 per group): a noninfected control; an infected, untreated control (normal saline solution); METR monotherapy at 7.5 or 15 mg/kg/day; AGS monotherapy at 10 or 15 mg/kg/day; and two combination‐therapy groups receiving METR 7.5 mg/kg/day alongside AGS at 10 or 15 mg/kg/day [14]. Infected groups were treated orally once daily for seven consecutive days. Sample size (*n* = 8 per group) was determined based on comparable previous murine giardiasis studies [9, 14–16] rather than an a priori power calculation, which is noted as a limitation. Although adequate to detect significant differences in primary parasitological outcomes, we acknowledge that larger cohorts may provide greater statistical power for molecular analyses. Based on previous in vivo studies demonstrating the biological activity of orally administered AGS at doses ranging from 10 to 30 mg/kg/day in mice, including beneficial effects on intestinal function and inflammatory responses, doses of 10 and 15 mg/kg/day were selected for the present study [17, 18]. In addition, the safety of these doses was evaluated in the current study by assessing hepatic toxicity in both healthy and *Giardia*‐infected mice. No evidence of hepatotoxicity or significant adverse effects on hepatic biochemical parameters was observed at either dose, further supporting the safety and appropriateness of the selected AS‐IV doses for the present experimental model. METR doses were selected according to previously established antigiardial experimental protocols and to investigate whether AGS could enhanced the efficacy of reduced‐dose METR therapy [13–16]. AGS and METR were freshly prepared prior to administration in sterile normal saline and were orally administered at a dosing volume of 10 mL/kg body weight. Each mouse was considered an independent biological replicate for statistical analysis. Technical replicates were averaged before inferential testing. No animals were excluded from the final analysis. Primary analytical variables included *Giardia* cyst number and cyst viability percentage. Secondary outcome variables included intestinal included intestinal secretory IgA (sIgA) concentrations, fold changes in inflammatory gene expression, serum electrolyte concentrations, and hepatic enzyme levels (Figure 1).

#### 2.4.4. Quantification of Parasitological Outcomes

Fecal samples were collected at the prespecified endpoint of 24 h after the final treatment dose and examined to determine cyst excretion levels. Viability assessment was performed using eosin staining, whereby nonviable cysts exhibited pink coloration, while viable cysts remained unstained [13]. Because the experimental protocol included euthanasia and tissue and serum collection at this prespecified 24‐h endpoint, longer‐term follow‐up assessments were not performed [13].

#### 2.4.5. Tissue Harvesting and Serum Collection

Following deep anesthesia induced by intraperitoneal administration of ketamine (100 mg/kg) and xylazine (10 mg/kg), mice were humanely euthanized. The duodenum was carefully excised, longitudinally opened, and processed according to the protocol described by Ghasemian Yadegari et al. [15]. Intestinal tissues were immediately snap‐frozen and stored at −80°C for subsequent molecular analyses. Blood samples were collected via cardiac puncture, centrifuged to obtain serum, and preserved for biochemical evaluations.

### 2.5. Biochemical and Electrolyte Analyses

Serum concentrations of sodium (Na^+^), potassium (K^+^), and chloride (Cl^-^) were determined using standard enzymatic, turbidimetric, and colorimetric methods, respectively. All measurements were performed using commercially available diagnostic kits (Pars Azmoon Co., Tehran, Iran) on an automated biochemistry analyzer following the manufacturer’s standardized protocols. The analytical sensitivity and detection ranges for each parameter were as follows: Na^+^ (range: 10–200 nmol/L), K^+^ (range: 0.5–10 nmol/L), and Cl^−^ (range: 10–200 nmol/L). Multilevel calibrators and quality control (QC) materials were included in each analytical run in accordance with standard clinical chemistry practice. All runs met acceptance criteria before sample data were reported. Intra‐ and inter‐assay CVs were maintained below 5% and 8%, respectively, across all biochemical parameters.

### 2.6. Assessment of Mucosal Immunity

The Mouse sIgA ELISA Kit (Elabscience, Wuhan, China; Cat. No. E‐EL‐M0420) was used to quantify intestinal sIgA concentrations. The assay sensitivity (minimum detectable concentration) was 0.094 ng/mL, with a dynamic detection range of 0.156–10 ng/mL. Prior to analysis, intestinal tissue homogenates were diluted 1:5 in the kit‐supplied sample diluent buffer to ensure that all sample readings fell within the linear range of the standard curve. A seven‐point recombinant protein standard curve (0.156, 0.313, 0.625, 1.25, 2.5, 5, and 10 ng/mL) was prepared and run in duplicate on each plate. High‐ and low‐concentration QCs, supplied by the manufacturer, were included in every assay run to verify inter‐assay reproducibility. Intra‐assay and inter‐assay coefficients of variation (CVs) were ≤ 8% and ≤ 12%, respectively, consistent with the manufacturer’s specifications. Optical density was measured at 450 nm using a microplate reader.

### 2.7. Inflammatory Gene Expression Analysis

#### 2.7.1. Tissue Processing and RNA Preparation

Duodenal tissues collected from experimental mice were subjected to total RNA isolation using a commercial extraction system (Total RNA Extraction Mini Kit Plus; Yekta Tajhiz Azma Co., Tehran, Iran, Cat No. YT2551). Extracted RNA samples were examined for purity and usability prior to enzymatic processing. To prevent amplification bias caused by residual genomic DNA, RNA preparations were enzymatically treated with RNase‐free DNase I (Fermentas, United States) before entering the reverse transcription workflow. RNA purity was evaluated spectrophotometrically using A260/A280 absorbance ratios, and only samples with ratios between 1.8 and 2.0 were included for downstream analyses.

#### 2.7.2. Reverse Transcription

Complementary DNA (cDNA) synthesis was performed using a commercial reverse transcription kit (YTA‐cDNA Synthesis Kit; Yekta Tajhiz Azma Co., Tehran, Iran, Cat No. YT4500), strictly following the manufacturer’s standardized protocol.

#### 2.7.3. Target Genes and Primer Design

Quantification focused on key inflammation‐related transcripts, including tumor necrosis factor‐alpha (*TNF-α*), nuclear factor kappa B (*NF-κB*), and interleukin‐1 beta (*IL-1β*). The gene‐specific primer sequences employed for amplification are detailed in Table 1 and were designed in accordance with the methodology described by Ghasemian Yadegari et al. [15]. Primer sequence validity was evaluated through in silico analysis against the National Center for Biotechnology Information (NCBI) reference database. In addition, primer specificity was independently verified using the BLAST alignment tool to exclude nontarget amplification. *β*‐Actin was used as the internal reference gene due to its stable expression across experimental groups. Amplification efficiency for all primer pairs ranged between 90% and 110% (calculated from standard curve slopes), the reference gene was validated for stable expression across experimental groups, and amplification specificity was confirmed by single‐peak melt‐curve analysis and agarose gel electrophoresis of PCR products.

**Table 1:** Primer sequences utilized in real‐time PCR analysis.

Primer	Sequence (5 ^′^–3 ^′^)	Amplicon size (bp)
IL‐1*β*	F: AACCTGCTGGTGTGTGACGTTC	256
	R: CAGCACGAGGCTTTTTTGTTGT	
TNF‐*α*	F: TGAACTTCGGGGTGATCGGT	182
	R: GGTGGTTTGTGAGTGTGAGGG	
NF‐*κ*B p65	F: AGGCAAGGAATAATGCTGTCCTG	209
	R: ATCATTCTCTAGTGTCTGGTTGG	
*β*‐actin	F: GTGACGTTGACATCCGTAAAGA	245
	R: GCCGGACTCATCGTACTCC	

*Note:* F, forward primer; R, reverse primer.

#### 2.7.4. Thermal Cycling Profile

PCR amplification was initiated with a primary denaturation phase at 96°C for 5 min. The reaction then proceeded through 35 cycles of denaturation at 96°C for 30 s and combined annealing/extension at 62°C for 60 s, followed by a terminal extension step at 73°C for 5 min.

#### 2.7.5. Computational Analysis

Transcript abundance was expressed as fold change relative to the uninfected control group, derived through application of the 2^−*ΔΔ*Ct^ algorithm. Prior to this calculation, raw Ct values were normalized against a stably expressed housekeeping gene *β*‐actin whose expression consistency across all groups had been pre‐verified using geNorm or NormFinder algorithms. Each target gene was amplified in independent triplicate reactions per sample, and mean Ct values were used as the basis for all subsequent calculations to minimize technical variability.

### 2.8. Toxicological and Safety Assessment

Serum concentrations of hepatic biomarkers, including alanine aminotransferase (ALT), aspartate aminotransferase (AST), and gamma‐glutamyl transferase (GGT), were quantified according to the IFCC (International Federation of Clinical Chemistry) recommended kinetic methods. All measurements were performed using commercially available diagnostic kits (Pars Azmoon Co., Tehran, Iran) on an automated biochemistry analyzer following the manufacturer’s standardized protocols. Serum samples were assayed undiluted, as recommended for these kits when samples fall within the expected physiological range; where sample values exceeded the upper limit of linearity, samples were diluted 1:2 with isotonic saline and re‐assayed. The analytical sensitivity and detection ranges for each parameter were as follows: ALT (range: 4–500 U/L), AST (range: 4–500 U/L), and GGT (range: 2–300 U/L) [16].

### 2.9. Statistical Analysis

Quantitative data are presented as mean ± standard deviation (SD). Normality of data distribution was assessed using the Shapiro–Wilk test prior to selection of parametric analyses. Intergroup differences were evaluated using one‐way analysis of variance (ANOVA), followed by Tukey’s honest significant difference (HSD) post hoc test for multiple pairwise comparisons. The comparisons between the infected untreated control and treatment groups were additionally verified using appropriate nonparametric alternatives (Kruskal–Wallis test followed by Dunn’s post hoc test) where normality assumptions were violated. All statistical analyses were performed using SPSS software (version 26.0; IBM Corp., Armonk, NY, United States). A two‐tailed *p* value of less than 0.05 was considered statistically significant. Exact *p* values, *η* ^2^ effect sizes, and 95% confidence intervals were calculated for principal outcome variables to strengthen quantitative interpretation of treatment effects. For qPCR analyses, statistical comparisons were performed using *Δ*Ct values, whereas fold changes were calculated using the 2^−*ΔΔ*Ct^ method for graphical presentation.

## 3. Results

### 3.1. In Vitro Antigiardial Effects of AGS Against *G. lamblia* Trophozoites

**Figure 2:**
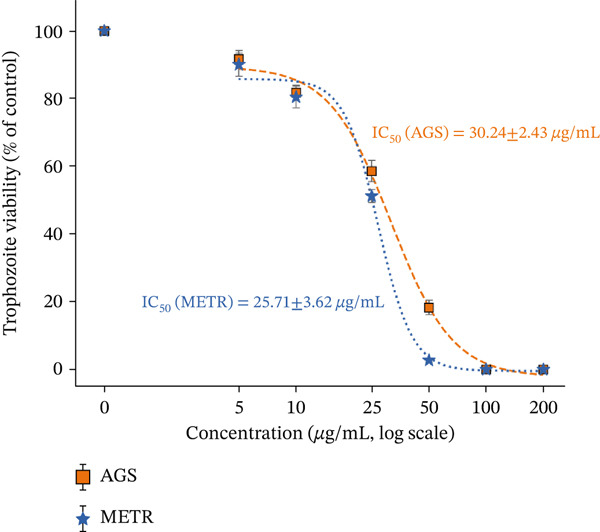
Concentration‐dependent effects of astragaloside IV (AGS) and metronidazole (METR) on Giardia lamblia trophozoite viability. Drug concentrations are presented on a logarithmic (log10) *x*‐axis, and the curves represent nonlinear dose–response regression. Data are expressed as mean ± standard deviation (SD) from three independent experiments (*n* = 3).

As presented in Figure 1, both AGS and METR exerted a clear concentration‐dependent inhibitory effect on the viability of *Giardia* trophozoites, with significantly greater inhibition observed at increasing concentrations compared with untreated cultures (*p* < 0.001). The calculated IC_50_ values were 30.24 ± 2.43 *μ*g/mL for AGS and 25.71 ± 3.62 *μ*g/mL for METR (Figure 2).

### 3.2. In Vivo Antigiardial Effects of AGS

Treatment of *Giardia*‐infected mice with METR, AGS, and their combinations produced pronounced, dose‐dependent reductions in both cyst load and viability (*p* < 0.001, Table 2). Mice receiving normal saline exhibited a high cyst count (728.2 ± 46.6) with almost complete viability (97.3*%* ± 2.5*%*), confirming robust infection establishment. AGS alone elicited moderate, dose‐dependent effects, with 10 mg/kg lowering cysts to 276.5 ± 27.3 and viability to 68.7*%* ± 7.9*%*, and 15 mg/kg reducing them to 162.3 ± 19.4 and 23.4*%* ± 3.5*%*, respectively (*p* = 0.0023 vs. control; *p* = 0.0081 15 mg/kg vs. 10 mg/kg). Notably, at the prespecified 24‐h post‐treatment endpoint, combination therapy with AGS (10 or 15 mg/kg) and METR 7.5 mg/kg resulted in no detectable cysts and viability (0.0 ± 0.0), reflecting greater short‐term antigiardial efficacy than either agent alone (*p* < 0.001 vs. all monotherapies and control). However, the persistence of this effect beyond the 24‐h assessment point was not evaluated.

**Table 2:** *Giardia lamblia*‐infected BALB/c mice: Parasitological outcomes. Dose‐dependent effects of metronidazole (METR), astragaloside (AGS), and combination therapy on cyst number and cyst viability (*n* = 8 per group).

Group	Treatment	Dose (mg/kg/day)	Cyst count (*m* *e* *a* *n* ± *S* *D*)	95% CI	Tukey	Viability (%)	95% CI	Tukey
**NI**	Noninfected control	—	—	—	—	—	—	—
**I**	Infected, untreated (saline)	—	728.2 ± 46.6	689.24–767.16	a	97.3 ± 2.5	95.21–99.39	a
**II**	METR 7.5 mg/kg	7.5	139.4 ± 12.8	128.70–150.10	d	31.3 ± 2.6	29.13–33.47	c
**III**	METR 15 mg/kg	15	5.6 ± 1.6	4.26–6.94	e	3.6 ± 2.3	1.68–5.52	d
**IV**	AGS 10 mg/kg	10	276.5 ± 27.3	253.67–299.33	b	68.7 ± 7.9	62.09–75.31	b
**V**	AGS 15 mg/kg	15	162.3 ± 19.4	146.08–178.52	c	23.4 ± 3.5	20.47–26.33	cd
**VI**	**AGS 10 + METR 7.5 mg/kg**	10 + 7.5	0.0 ± 0.0	0.00–0.00	**f**	0.0 ± 0.0	0.00–0.00	**e**
**VII**	**AGS 15 + METR 7.5 mg/kg**	15 + 7.5	0.0 ± 0.0	0.00–0.00	**f**	0.0 ± 0.0	0.00–0.00	**e**

*Note:* Values are presented as mean ± SD (*n* = 8 per group). 95% confidence intervals (CIs) calculated using t‐distribution (df = 7, *t* = 2.365). Different lowercase letters (a, b, c…) indicate statistically significant differences between groups based on Tukey’s HSD post hoc test following one‐way ANOVA (*p* < 0.05); groups sharing the same letter are not significantly different. Bold rows (Groups VI–VII) indicate combination therapy achieving absence of detectable cyst shedding at 24 h post‐treatment.

Abbreviations: AGS: astragaloside IV; METR: metronidazole; NI: noninfected control.

### 3.3. Electrolyte and Biochemical Outcomes

*Giardia* infection induced a profound decrease in all measured electrolytes, with the saline‐treated infected group showing Na^+^ 72.2 ± 9.7 nmol/L, K^+^ 1.63 ± 0.35 nmol/L, and Cl^−^ 52.6 ± 0.35 nmol/L (*p* < 0.001 vs. noninfected controls). AGS monotherapy produced moderate improvement in a dose‐dependent manner: 10 mg/kg achieved partial correction, and 15 mg/kg further improved electrolyte levels, though values remained below those of noninfected mice (*p* = 0.032). Notably, AGS + METR 7.5 mg/kg exerted an enhanced efficacy of the combination compared with either agent alone, fully restoring and slightly exceeding normal electrolyte levels (AGS 10 mg/kg + METR 7.5 mg/kg: Na^+^ 156.3 ± 15.6 nmol/L, K^+^ 5.3 ± 0.42 nmol/L, Cl^−^ 116.2 ± 11.4 nmol/L; AGS 15 mg/kg + METR 7.5 mg/kg: Na^+^ 162.2 ± 19.4 nmol/L, K^+^5.59 ± 0.61 nmol/L, Cl^−^114.5 ± 8.8 nmol/L; *p* = 0.0069 vs. single treatments and saline) (Table 3).

**Table 3:** Comparison the effects of metronidazole (METR), astragaloside (AGS), and their combinations on the serum level of sodium, potassium, and chloride in the *Giardia*‐infected mice (*n* = 8 per group).

Group	Treatment	Dose (mg/kg/day)	Na^+^ (nmol/L)	95% CI	Tukey	K^+^ (nmol/L)	95% CI	Tukey	Cl^-^ (nmol/L)	95% CI	Tukey
**NI**	Noninfected control	—	141.2 ± 12.9	130.41–151.99	d	4.96 ± 0.67	4.40–5.52	c	106.2 ± 12.3	95.92–116.48	c
**I**	Infected, untreated (saline)	—	72.2 ± 9.7	64.09–80.31	a	1.63 ± 0.35	1.34–1.92	a	52.6 ± 0.35	52.31–52.89	a
**II**	METR 7.5 mg/kg	7.5	106.6 ± 12.3	96.32–116.88	Bc	2.70 ± 0.31	2.44–2.96	b	82.4 ± 7.6	76.05–88.75	b
**III**	METR 15 mg/kg	15	139.7 ± 13.2	128.66–150.74	d	4.90 ± 0.51	4.47–5.33	c	93.2 ± 9.4	85.34–101.06	bc
**IV**	AGS 10 mg/kg	10	97.3 ± 8.6	90.11–104.49	b	2.30 ± 0.21	2.12–2.48	b	84.1 ± 9.1	76.49–91.71	b
**V**	AGS 15 mg/kg	15	112.7 ± 10.2	104.17–121.23	c	3.10 ± 0.20	2.93–3.27	b	92.7 ± 8.2	85.84–99.56	bc
**VI**	**AGS 10 + METR 7.5 mg/kg**	10 + 7.5	156.3 ± 15.6	143.26–169.34	**e**	5.30 ± 0.42	4.95–5.65	**c**	116.2 ± 11.4	106.67–125.73	**d**
**VII**	**AGS 15 + METR 7.5 mg/kg**	15 + 7.5	162.2 ± 19.4	145.98–178.42	**e**	5.59 ± 0.61	5.08–6.10	**c**	114.5 ± 8.8	107.14–121.86	**d**

*Note:* Values are presented as mean ± SD (*n* = 8 per group). 95% confidence intervals (CIs) calculated using t‐distribution (df = 7, t = 2.365). Different lowercase letters (a, b, c…) indicate statistically significant differences between groups based on Tukey’s HSD post hoc test following one‐way ANOVA (*p* < 0.05); groups sharing the same letter are not significantly different. Bold rows (Groups VI–VII) indicate combination therapy achieving absence of detectable cyst shedding at 24 h post‐treatment.

Abbreviations: AGS: astragaloside; Cl^−^: chloride; K^+^: potassium; METR: metronidazole; Na^+^: sodium; NI: noninfected control.

### 3.4. Mucosal Immunological Outcomes

**Figure 3:**
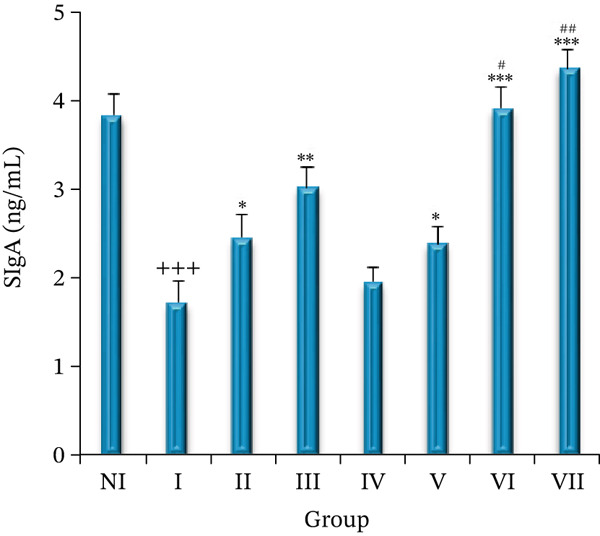
Effects of astragaloside (AGS), metronidazole (METR), and their combinations on intestinal secretory immunoglobulin A (sIgA) levels in *Giardia lamblia*‐infected BALB/c mice. Data are presented as mean ± SD (*n* = 8 per group). Different lowercase letters (a–e) indicate significant differences among groups (one‐way ANOVA followed by Tukey’s HSD test, *p* < 0.05); groups sharing the same letter are not significantly different. NI: noninfected control; I: infected‐untreated control; II: METR 7.5 mg/kg; III: METR 15 mg/kg; IV: AGS 10 mg/kg; V: AGS 15 mg/kg; VI: METR 7.5 + AGS 10 mg/kg; VII: METR 7.5 + AGS 15 mg/kg. +++*p* < 0.001 vs. NI; ∗*p* < 0.05, ∗∗*p* < 0.01, ∗∗∗*p* < 0.001 vs. I; #*p* < 0.05, ##*p* < 0.01 vs. III.

Based on ELISA quantification, *Giardia*‐infected mice receiving normal saline exhibited the lowest sIgA level (1.73 ng/mL), which was significantly lower than all treatment groups (*p* < 0.001), indicating severe impairment of mucosal humoral immunity. The administration of AGS alone produced a moderate elevation of sIgA, reaching 1.96 ng/mL at 10 mg/kg (*p* = 0.027 vs. saline) and 2.38 ng/mL at 15 mg/kg (*p* = 0.0073 vs. saline); however, both AGS monotherapy groups remained significantly lower than the high‐dose METR group (*p* = 0.046). Notably, combination therapy with AGS and METR induced the most pronounced enhancement of mucosal IgA secretion, with sIgA concentrations increasing to 3.91 and 4.36 ng/mL, respectively. These values were significantly higher than all monotherapy groups (*p* < 0.001) and exceeded even the high‐dose METR group (*p* = 0.0035), demonstrating an enhanced combination efficacy on intestinal immune restoration (Figure 3).

### 3.5. Inflammatory Gene Expression

**Figure 4:**
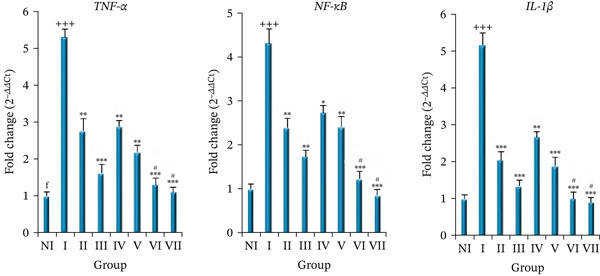
Effects of astragaloside (AGS), metronidazole (METR), and their combinations on the expression of pro‐inflammatory genes in *Giardia lamblia*‐infected BALB/c mice. Panels show relative expression levels of tumor necrosis factor‐alpha (TNF‐*α*), nuclear factor kappa B (NF‐*κ*B), and interleukin‐1 beta (IL‐1*β*). Data are presented as mean ± SD (*n* = 8 per group). Different lowercase letters (a–f) indicate significant differences among groups (one‐way ANOVA followed by Tukey’s HSD test, *p* < 0.05); groups sharing the same letter are not significantly different. NI: noninfected control; I: infected‐untreated control; II: METR 7.5 mg/kg; III: METR 15 mg/kg; IV: AGS 10 mg/kg; V: AGS 15 mg/kg; VI: METR 7.5 + AGS 10 mg/kg; VII: METR 7.5 + AGS 15 mg/kg. +++*p* < 0.001 vs. NI; ∗*p* < 0.05, ∗∗*p* < 0.01, ∗∗∗*p* < 0.001 vs. I; #*p* < 0.05 vs. III.

The evaluation of key inflammatory mediators demonstrated significant modulation of pro‐inflammatory signaling pathways across the experimental groups (one‐way ANOVA, *p* < 0.001). *Giardia*‐infected mice treated with normal saline exhibited the highest expression levels of *TNF-α* (5.3‐fold change), *NF-κB* (4.29‐fold change), and *IL-1β* (5.16‐fold change), reflecting a pronounced inflammatory state associated with active infection and mucosal tissue damage (Figure 4). The administration of AGS alone also attenuated inflammatory marker expression in a dose‐dependent manner, with moderate but significant reductions at 10 mg/kg (*p* = 0.023) and stronger effects at 15 mg/kg (*p* = 0.0052), although these levels remained significantly higher than those observed in the high‐dose METR group (*p* = 0.039). Notably, combination therapy with AGS at 10 and 15 mg/kg and METR produced the most profound suppression of inflammatory signaling, reducing *TNF-α*, *NF-κB*, and *IL-1β* expression to near‐baseline levels (1.31 to 1.01‐fold change and 1.11 to 0.91‐fold change). These values were significantly lower than all monotherapy groups (*p* < 0.001), indicating an enhanced anti‐inflammatory effect.

### 3.6. Biochemical and Hepatoprotective Effects

**Figure 5:**
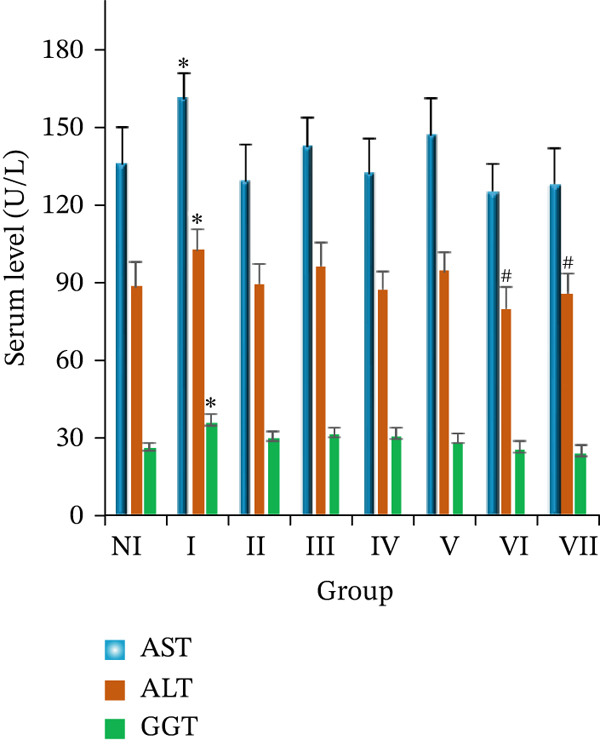
Effects of astragaloside (AGS), metronidazole (METR), and their combination on serum levels of alanine aminotransferase (ALT), aspartate aminotransferase (AST), and gamma‐glutamyl transferase (GGT) in *Giardia*‐infected mice. Data are presented for each treatment group (*n* = 8 per group). NI: noninfected control; I: infected‐untreated control. II: METR 7.5 mg/kg/day; III: METR 15 mg/kg/day; IV: AGS 10 mg/kg/day; V: AGS 15 mg/kg/day; VI: AGS 10 mg/kg/day + METR 7.5 mg/kg/day; VII: AGS 15 mg/kg/day + METR 7.5 mg/kg/day. ∗*p* < 0.05 vs. NI; #*p* < 0.05 vs. I.

Evaluation of hepatic function revealed that *Giardia* infection induced mild liver stress, as shown by elevated serum AST, ALT, and GGT levels in untreated mice (AST: 161.2 vs. 136.1 U/L; ALT: 102.4 vs. 88.3 U/L; GGT: 35.6 vs. 26.2 U/L, *p* = 0.016). The administration of METR (7.5–15 mg/kg) or AGS (10–15 mg/kg) individually did not result in additional hepatotoxicity and moderately improved enzyme profiles (AST: 129.4–147.2 U/L; ALT: 87.5–96.4 U/L; GGT: 28.7–31.2 U/L). Notably, the combination of AGS 15 mg/kg with METR 7.5 mg/kg was both safe and highly effective, restoring liver enzyme levels nearly to baseline (AST: 125.4–128.3 U/L; ALT: 79.8–85.7 U/L; GGT: 23.9–25.4 U/L) (Figure 5).

## 4. Discussion

Giardiasis is conventionally managed with nitroimidazole and nitrofuran derivatives, principally METR and furazolidone [4], but their inconsistent efficacy and adverse‐effect profile continue to limit patient adherence [5]. Given the broad biological activity of AGS and the ongoing need for safer, more effective antigiardial agents, this study evaluated the antigiardial efficacy of AGS and its interaction with METR in a murine model of *G. lamblia* infection.

The marked reduction in cyst burden after AGS treatment, especially in combination with reduced‐dose METR, points to a dual mechanism rather than a purely host‐mediated one. Prior literature on AGS and *Astragalus* derivatives has focused almost exclusively on immunoregulatory, antioxidant, and anti‐inflammatory activity [9–11], with no direct evidence of antiparasitic action against protozoa such as *G. lamblia*, *T. gondii*, or *Leishmania* spp. Indirect support comes from whole‐plant *Astragalus* extracts: chloroform extracts of *A. maximus* reduce *G. lamblia* cyst and trophozoite viability in vitro, apparently by disrupting membrane integrity and triggering apoptotic pathways [12], and *Astragalus* extracts likewise suppress intracellular *T. gondii* replication via enhanced nitric oxide and interferon‐*γ* production [19]. However, these effects were attributed to complex phytochemical mixtures rather than an isolated compound. To test whether AGS itself has intrinsic antigiardial activity, we performed in vitro susceptibility assays on trophozoites and found a concentration‐dependent reduction in viability (IC_50_ = 30.24 *μ*g/mL). These findings indicate that AGS exhibits direct antigiardial activity against *G. lamblia* trophozoites in vitro. However, the relative contributions of direct antigiardial activity and host‐directed immunomodulation to the reduced cyst shedding observed in vivo could not be determined in the present study.

Secretory IgA is the principal mucosal defense against *G. lamblia*, limiting trophozoite attachment, motility, and persistence in the gut lumen, where the parasite remains confined without invading host tissue [20, 21]. Impaired IgA responses are associated with prolonged infection and chronicity [22]. Consistent with this, untreated infected mice showed reduced sIgA levels, likely reflecting epithelial disruption and impaired plasma cell activity. AGS treatment restored sIgA levels, suggesting recovery of mucosal immune competence, and the superior effect of combination therapy likely reflects both reduced antigenic burden from parasite clearance and direct support of plasma cell and epithelial IgA secretion. Restoring sIgA responses may therefore be a valuable therapeutic target, supporting combined pharmacologic–immunomodulatory approaches to giardiasis management.

AGS treatment also normalized *TNF-α*, *NF-κB*, and *IL-1β* levels, consistent with reports that *Giardia* activates innate immune signaling in epithelial and immune cells [23], with *TNF-α* contributing to barrier impairment and *NF-κB* driving downstream cytokine expression [24]. Combination therapy restored these markers to near‐baseline levels most effectively, likely reflecting reduced antigenic stimulation from parasite clearance together with direct suppression of epithelial NF‐*κ*B activation. These findings support a strategy of concurrently targeting the parasite and host inflammatory response, and position AGS as a candidate immunomodulatory adjunct to standard antigiardial therapy. Beyond cytokine suppression, AGS may also influence epithelial permeability, motility, mucosal drug absorption, or local METR bioavailability, and could affect the gut microenvironment or mucus barrier; the present design cannot distinguish these pharmacokinetic, epithelial, microbiome‐related, and immunological contributions, and future studies including pharmacokinetic and intestinal transport analyses, and evaluation of additional pathways such as MAPK and NLRP3 inflammasome signaling [25] are needed to resolve them.

Improved hepatic biochemical markers after AGS treatment align with its known hepatoprotective and antioxidant effects in other injury models. In alcohol‐induced hepatotoxicity, oral AGS (50–500 mg/kg) attenuates transaminase elevation and oxidative damage via modulation of the gut–liver axis and inflammasome inhibition [26]; in diet‐induced fatty liver disease, AGS (20–80 mg/kg) improves histopathology and normalizes enzyme levels through downregulation of TLR4/MyD88/NF‐*κ*B signaling [27]. These findings suggest AGS may also mitigate systemic inflammatory sequelae of intestinal giardiasis.

This study has several strengths: to our knowledge, it is the first evaluation of purified AGS in an *in vivo* murine giardiasis model; it integrates parasitological, immunological, molecular, biochemical, and hepatoprotective endpoints; and it shows that AGS can enhance reduced‐dose METR while preserving a favorable biochemical safety profile, supporting its translational potential as an adjunctive immunomodulatory agent. The superior efficacy observed with the AGS–METR combination is consistent with recent experimental evidence indicating that natural products may enhance the therapeutic efficacy of METR against Giardia infection. For example, combination treatment with black tea and METR achieved complete parasite clearance in experimentally infected mice [28].

### 4.1. Mechanistic Considerations

**Figure 6:**
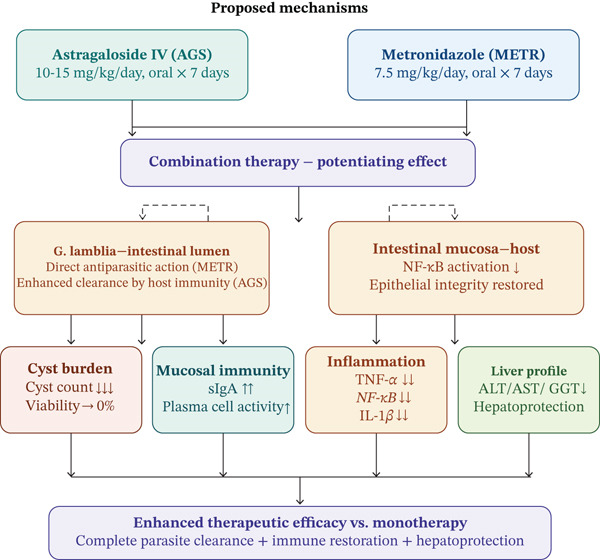
Proposed mechanisms underlying the effects of Astragaloside IV (AGS) combined with metronidazole (METR) in *Giardia lamblia*‐infected BALB/c mice. The schematic summarizes the proposed immunomodulatory effects of AGS, including restoration of intestinal secretory immunoglobulin A (sIgA), suppression of pro‐inflammatory signaling (NF‐*κ*B, TNF‐*α*, and IL‐1*β*), and improvement of infection‐associated hepatic alterations (ALT, AST, and GGT), together with the antigiardial activity of METR. This schematic represents a proposed and hypothetical mechanism based on the present findings; the depicted pathways and interactions were not experimentally verified in this study. AGS: Astragaloside IV; ALT: alanine aminotransferase; AST: aspartate aminotransferase; GGT: gamma‐glutamyl transferase; IL‐1*β*: interleukin‐1 beta; METR: metronidazole; sIgA: secretory immunoglobulin A; NF‐*κ*B: nuclear factor kappa B; TNF‐*α*: tumor necrosis factor‐alpha; ↑: increase; ↓: decrease.

The concurrent reduction in cyst shedding and modulation of sIgA, *TNF-α*, *NF-κB*, and *IL-1β* is consistent with an immunomodulatory contribution of AGS; however, no experiment directly established causality between these pathways and the absence of detectable cyst shedding at 24 h post‐treatment. The proposed mechanism (Figure 6) should therefore be regarded as a working hypothesis rather than an established biological process.

### 4.2. Limitations and Future Directions

Pharmacokinetic properties of AGS—systemic exposure, tissue distribution, mucosal concentration, and interaction with METR—were not assessed, limiting extrapolation to clinical dosing. Although in vitro assays confirmed direct antigiardial activity, the relative contribution of direct versus host‐mediated effects in vivo could not be quantitatively partitioned; studies using immunodeficient models could resolve this. The design used a single murine model and *Giardia* isolate, limiting generalizability across assemblages and host species. Importantly, parasitological outcomes were assessed only at the prespecified 24‐h post‐treatment endpoint. Consequently, the present study cannot determine whether the absence of detectable cyst shedding in the combination groups represents sustained parasite clearance, transient suppression of shedding, or potential recurrence after treatment cessation. Longitudinal assessments at 48 h, 72 h, and 7 days after treatment would be required to establish the durability of the observed antigiardial effect and to evaluate possible relapse. These follow‐up assessments should therefore be incorporated into future studies. The inflammatory panel was restricted to TNF‐*α*, NF‐*κ*B, and IL‐1*β*; broader profiling (IL‐6, IL‐10, IFN‐*γ*, TGF‐*β*, Th17, and regulatory T‐cell pathways) would clarify the immunomodulatory mechanism further. Finally, although combination therapy outperformed monotherapy, formal synergy analyses (Combination Index, Bliss independence, or Loewe additivity) were not conducted; the present findings should therefore be interpreted as evidence of enhanced combination efficacy rather than confirmed pharmacological synergism.

### 4.3. Translational Implications

Strategies that reduce METR exposure while preserving efficacy through adjunctive immunomodulation could improve tolerability and mitigate dose‐related toxicity. These findings extend the existing literature on the anti‐inflammatory and immunomodulatory properties of AGS by providing in vivo evidence of its potential as a host‐directed adjunct in experimental giardiasis.

## 5. Conclusion

In summary, AGS IV administration was associated with a reduced *Giardia* cyst burden at the prespecified 24‐h post‐treatment endpoint, restoration of mucosal immune responses, attenuation of inflammatory signaling, and improved biochemical profiles in experimentally infected mice. These findings suggest that AGS IV may function as an immunomodulatory and anti‐inflammatory adjunct and may contribute to improved short‐term treatment outcomes when combined with METR. However, the durability of parasite clearance, the potential for recurrence after treatment cessation, and formal pharmacodynamic synergistic interactions were not evaluated in the present study. Further longitudinal and mechanistic studies are warranted to determine the persistence and mechanisms of the observed effects.

## Author Contributions

Javad Ghaemian Yadegari and Hossein Mahmoudvand were responsible for the design and supervision of the experimental procedures. Sahar Mahmoudvand, Aram Oladi, Amal Khudair Khalaf, and Ahmad Adineh conducted the experiments and gathered the data. The primary manuscript was drafted by Hossein Mahmoudvand and Javad Ghaemian Yadegari.

## Funding

No funding was received for this manuscript.

## Disclosure

All authors have critically reviewed and approved the final version of the manuscript for submission.

## Consent

The authors have nothing to report.

## Conflicts of Interest

The authors declare no conflicts of interest.

## Data Availability

The datasets generated and/or analyzed during the current study are available from the corresponding author upon reasonable request.
